# Hypersensitive Detection and Quantitation of BoNT/A by IgY Antibody against Substrate Linear-Peptide

**DOI:** 10.1371/journal.pone.0058908

**Published:** 2013-03-21

**Authors:** Tao Li, Hao Liu, Kun Cai, Maoren Tian, Qin Wang, Jing Shi, Xiang Gao, Hui Wang

**Affiliations:** State Key Laboratory of Pathogens and Biosecurity, Beijing Institute of Microbiology and Epidemiology, Fengtai District, Beijing, PR China; Beijing Institute of Microbiology and Epidemiology, China

## Abstract

Botulinum neurotoxin A (BoNT/A), the most acutely poisonous substance to humans known, cleave its SNAP-25 substrate with high specificity. Based on the endopeptidase activity, different methods have been developed to detect BoNT/A, but most lack ideal reproducibility or sensitivity, or suffer from long-term or unwanted interferences. In this study, we developed a simple method to detect and quantitate trace amounts of botulinum neurotoxin A using the IgY antibody against a linear-peptide substrate. The effects of reaction buffer, time, and temperature were analyzed and optimized. When the optimized assay was used to detect BoNT/A, the limit of detection of the assay was 0.01 mouse LD_50_ (0.04 pg), and the limit of quantitation was 0.12 mouse LD_50_/ml (0.48 pg). The findings also showed favorable specificity of detecting BoNT/A. When used to detect BoNT/A in milk or human serum, the proposed assay exhibited good quantitative accuracy (88% < recovery < 111%; inter- and intra-assay CVs < 18%). This method of detection took less than 3 h to complete, indicating that it can be a valuable method of detecting BoNT/A in food or clinical diagnosis.

## Introduction

Botulinum neurotoxin (BoNT), the most acutely toxic substance to humans known, is produced by *Clostridium botulinum* under anaerobic conditions [1], [2]. Seven types or serotypes (A to G) of botulinum toxin are currently known. Each serotype is composed of a heavy and a light chain linked by disulfide bonds [3], [4]. The heavy chain is responsible for binding to specific pre-synaptic neuronal cell receptors and facilitating internalization. The light chain is a zinc-dependent endopeptidase that specifically cleaves soluble SNARE proteins essential for docking and fusion neurotransmitters containing vesicles at the nerve terminal. Types A, E, and C1 toxins cleave SNAP25_1-206_ (synaptosomal associated protein with 25 kDa molecular mass) at the Q197–R198, R180–I181, and R198–A199 positions [5]. Types B, D, F, and G toxins cleave vesicle-associated membrane protein (VAMP) at the Q76–F77, K59–L60, Q58–K59, and A81–A82 positions [6]. Types C1 toxin is known to also cleave Syntaxin.

Botulinum neurotoxins type A (BoNT/A) is the most toxic serotype to human. The 50% lethal dose (LD_50_) of BoNT/A to humans is only 0.1–1 ng/kg [2]. Given its small intoxicating dose, short eclipse period, and simple production, BoNT/A is a potential bioterrorism agent. Thus, BoNT/A has become a research hotspot in medical shielding research in recent years. If botulism diagnosis is promptly made, a proper therapy method can be applied and significantly decrease fatality. Therefore, a swift, precise assay for botulinum neurotoxin analysis is important for BoNT prevention and cure.

The mouse bioassay has been the standard for testing BoNT-containing samples for the past 30 years [7]–[9]. However, this assay is time consuming, requires the use of many animals, and has poor repeatability because of numerous fluctuant parameters involved. Several in vitro assays have also been reported for the detection of BoNT/A, relying either on mass spectrometry [10]–[12], immunological detection [13]–[15], Förster resonance energy transfer (FRET) [16]–[18], or endopeptidase activity [19]–[27]. The advantage of the endopeptidase assay is that it measures and quantitates the L-chain activity of the toxin, which is directly responsible for neurotransmission inhibition. However, many of these methods require a multi-step procedure or suffer from high variability, low sensitivity, or long reaction time.

The residues of the substrate SNAP25 at cleavage sites, which are normally buried in a helix, are exposed after BoNT cleavage, making the substrate become a linear peptide. Using the IgY antibody against this linear-peptide substrate, we improved a previous endopeptidase assay and developed a simple method for the detection and quantitation of BoNT/A. This method can be expanded to detect other types of botulinum toxins or specific enzymes in the future.

## Materials and Methods

### Bacterial strains, plasmids, and media

*C. botulinum* type A expression vectors pET32a (+) and pET22b (+) were from in our laboratory. *Escherichia coli* BL21 (DE3), *E. coli* DH5a, and pMD18-T cloning vectors were purchased from Beijing TransGen Biotech (China). Taq DNA polymerase, T4 DNA ligase, and restriction endonucleases were obtained from New England Biolabs (Beijing, China). PCR primers were synthesized by Beijing Sunbio Tech Co. Ltd. Plasmid mini-kits and gel extraction kits were from Beijing Biomed Co. Ltd. HisTrap FF columns (5 mL) were purchased from GE Healthcare (Beijing, China). All other chemicals and reagents were obtained from other commercial sources and were of the highest purity available.

### Animals and ethics statement

All necessary permits were obtained for the animal experiments. Approval of the Institutional Ethics Review Committee of Beijing Institute of Microbiology and Epidemiology, China was also obtained. All procedures on the animals were carried out in strict accordance with the regulations of the Beijing Institute of Microbiology and Epidemiology Animal Care and Use Committee (2009-07-20). Leghorns (19 weeks old) and BALB/c mice (weighing 20±2 g) were purchased from the Laboratory Animal Center of the Academy of Military Medical Sciences, Beijing, China. The animals were fed with standard diet and water, maintained under the following conditions: 12 h light/12 h dark controlled lighting, 24 °C to 28 °C temperature, and 55% relative humidity. All animals were handled under the care and supervision of a veterinarian. The mice, which were severely injured by high dose of toxin injected, were sacrificed by cervical dislocation at the end of experiment of LD50 test. The immunized leghorns laid eggs until their natural death.

### Expression and purification of SNAP25 and BoNT/A light chain (ALc)

The gene of SNAP25 was obtained by RT-PCR from the mouse brain tissue RNA. The PCR product was ligated into pMD18-T and transformed into an *E. coli* strain DH5α. The isolated cloning plasmid and the pET22b (+) vector were digested and ligated to form the expression plasmid pET22b-SNAP25. The pET22b-SNAP25 was transformed into *E. coli* strain BL21 (DE3). When the *E. coli* cells grew to the mid-exponential phase (OD_600_, 0.4–0.6), the cultures were induced with 1 mM IPTG, and incubated for additional 4 h. The concentrated bacterial suspensions were disrupted by mild sonication. The lysate was centrifuged, and the supernatant fraction containing the required proteins was retained for further purification. Purification was performed following the instructions of the Ni-column Affinity Chromatography using a Pharmacia fast protein liquid chromatography system. The expression and purified ALc was similar to SNAP25, except using the expression vector pET32a (+).

### Synthetic peptide substrate for BoNT/A (subA) and IgY antibody

A 10 a.a. peptide subA:NKTRIDEANQ located N-terminal of the cleavage site of BoNT/A at SNAP25 was synthesized. To prepare anti-subA IgY samples, the synthesized peptide was mixed with KLH at the amino terminal to improve the antigenicity, and then emulsified with an equal volume of complete Freund’s adjuvant or incomplete Freund’s adjuvant. The 19-week-old specific-pathogen-free leghorns were then immunized by injecting the emulsion into 4–6 sites at the pectoral muscle of each hen, and then boosted on weeks 2, 5, and 11. Eggs were collected, labeled accordingly, and stored at 4 °C until use.

IgY was obtained using the water dilution method under acidic conditions, using a previously described method (Wang et al., 2010), with modification. The purified IgY concentration was estimated by the Bradford method and finally identified by SDS–PAGE. The IgY titer and specificity were detected by ELISA.

### Production and identification of BoNT/A and BoNT/E

The bacteria strain used for production of BoNT/A and BoNT/E were *C. botulinum* strain 230611 and 230612 which were isolated from cases of food-borne botulism in China. *C. botulinum* strain 230611 and 230612 was grown at 37 °C (30°C) in TPGY medium. After 48 h of incubation, the culture was processed for toxin purification using a previously described method [28], with modifications. The extracted toxins serially diluted with sodium phosphate buffer were assayed for toxicity by intraperitoneal injection into groups of 10 BALB/c mice (Laboratory Animal Center, Beijing, China). The concentration that killed half of the animals within 7 days was considered as the LD_50_. The extracted BoNT/A was mixed with a half-volume of enzyme reaction buffer (50 mM HEPES, 2 mM DTT, 10 µM ZnCl_2_, pH 7.5) and incubated at 37 °C for 15 min. Purified SNAP25 proteins were then added, and the mixture was incubated at 37 °C for 1 h. The endopeptidase reaction was analyzed by SDS-PAGE.

### Optimization of reaction buffer of the endopeptidase assay

To determine the optimized condition of the endopeptidase assay, an ALc endopeptidase assay was performed on a SNAP25coated plate with three different reaction buffers. First was Ni-NTA buffer (B buffer; pH 7.4), containing 0.02 M Na_3_PO_4_, 0.5 M NaCl, and 0.4 M imidazole. Second was PBS buffer (pH 7.2), containing 0.008 M NaH_2_PO_4_, 0.002 M Na_2_HPO_4_, and 0.145 M NaCl. Third was PB buffer (pH 7.2), containing 0.008 M NaH_2_PO_4_ and 0.002 M Na_2_HPO_4_.

The purified recombinant proteins (ALc and SNAP25) in B buffer were dialyzed to PBS and PB buffers at 4 °C for 24 h. Plates were coated with SNAP25 in three buffers (PBS buffer, B buffer, and H_2_O) at 0.4 µg/well, and incubated overnight at 4 °. The plates were washed three times at 3 min intervals with PBST, and then blocked with BSA at 37 °C for 2 h. The ALc proteins in the three buffers (PBS, PB, and B buffers) were mixed with a half-volume of reaction buffer (50 mM HEPES, 2 mM DTT, 10 µM ZnCl_2_, pH 7.5). Then, serially diluted mixtures were added (100 µL/well), maintaining the temperature at 37 °C for 1 h. The samples were washed, and anti-subA IgY was added at 200 µL/well and incubated at 37 °C for 1 h. The samples were rewashed and horseradish-peroxidase conjugated rabbit anti-hen secondary antibody (Sigma Chemical Co.) was added and incubated at 37 °C for 0.5 h. Then, AB buffer was added and the mixture was incubated for 10 min. The reaction was stopped with 2 M H_2_SO_4_. The result was read at 450 nm using an absorbance microplate reader. A reading two times higher than that of the negative control was regarded as positive.

### Optimization of reaction temperature and time of the endopeptidase assay

To determine the optimum reaction temperature and time of the ALc endopeptidase assay, gradient-diluted ALc was added to a SNAP25 coated plate and incubated at two reaction temperatures (37 °C and RT) for three reaction times (1, 2, and 4 h) with an optimized reaction buffer combination, and then tested by ELISA. The result was read at 450 nm using an absorbance microplate reader. A reading two times higher than that of the negative control was regarded as positive.

### Detection and quantitation of BoNT/A and BoNT/E standard protein using IgY antibody

To determine sensitivity and specificity, serially diluted BoNT/A and BoNT/E proteins were added to a SNAP25 coated plate, incubated under the optimized condition, and tested by ELISA for parallel line analysis. The limit of detection (LOD) and limit of quantitation (LOQ) of the method were detected by BoNT/A. The specificity of the method was verified by BoNT/E.

A standard curve was constructed by plotting the absorbance values (mean of triplicate wells) against standard toxin concentrations. Unknown concentrations were determined from the linear regression equation.

### Calculation of method precision by testing BoNT/A mixed with milk and human serum

Three BoNT/A concentrations (0.24 LD_50_, 0.32 LD_50_, and 0.48 LD_50_) were mixed with reaction buffer, milk, or human serum, and then detected using IgY antibody. The ELISA result was read at 450 nm by an absorbance microplate reader. Intra- and inter-assay precisions were analyzed by BoNT/A incorporated in milk and human serum.

### Statistical analysis

The results are presented as the mean ± standard error (SE). Statistical significance was determined by Student’s t test or the chi-squared test. *P* values <0.05 were considered significant.

## Results

### Expression and characterization of SNAP25 and BoNT/ALc

**Figure 1:**
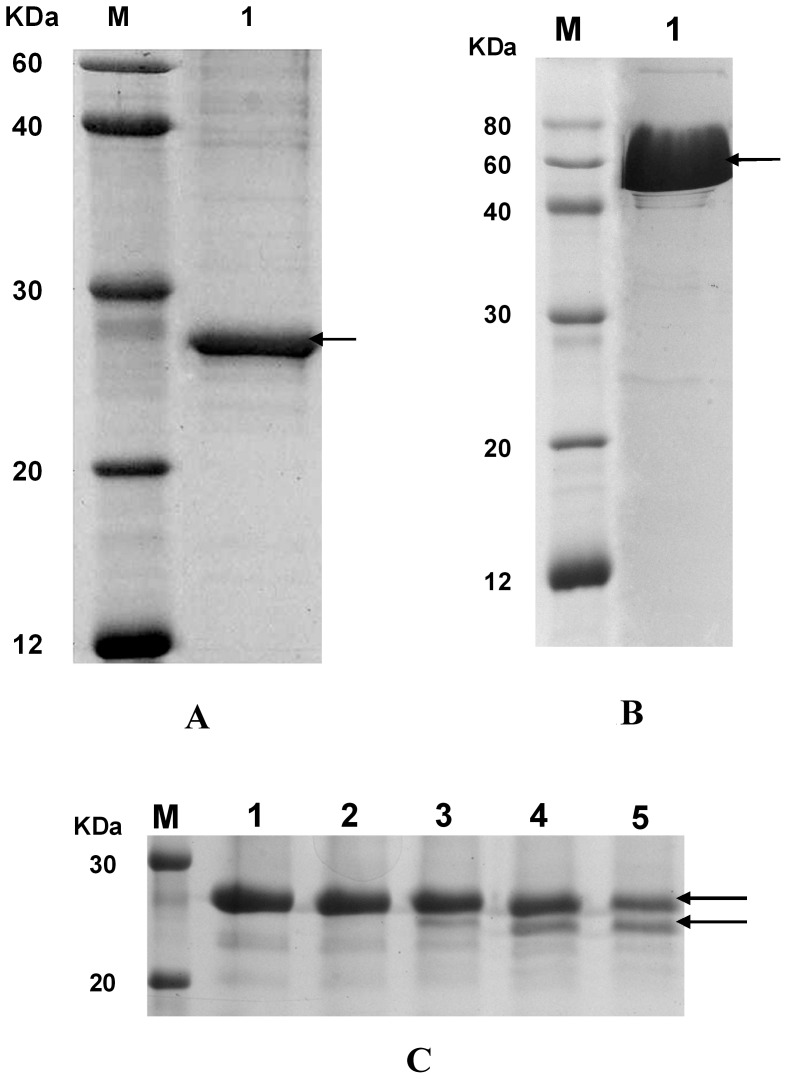
SDS-PAGE results of purified SNAP25 and ALc, as well as cleaved SNAP25 by BoNT/A and ALc. A) SDS-PAGE result of purified SNAP25. M: marker; 1: purified SNAP25. B) SDS-PAGE result of purified ALc. M: marker; 1: purified ALc. C) SDS-PAGE result of purified SNAP25 mixed with ALc. M: marker; 1: 10 µl of SNAP25; 2: 10 µl of SNAP25 + 5 µl of ALc; 3: 10 µl of SNAP25 + 10 µl of ALc; 4: 10 µl of SNAP25 + 15 µl of ALc; 5: 10 µl of SNAP25 + 20 µl of ALc.

The expression and purity of SNAP25 and ALc were verified by SDS-PAGE and visualized by Coomassie blue staining and Western blot analysis using mouse anti-His-tag antibody and horse anti-BoNT/A antibody. The purity of the obtained proteins was verified as 85% by one-step Ni-column purification analyzed by the Bandscan 5.0 software. The molecular weights of the proteins matched the theoretically predicted ones, i.e., 28 and 60 kDa (Figs. 1A and 1B). The final yields of purified SNAP25 and ALc were 7.76 and 30.01 mg/L culture. Purified ALc protein was used to cleave recombinant SNAP25. The result indicated that recombinant ALc had an endopeptidase toward SNAP25 and presented linear concentration–response relations (Fig. 1C).

The LD_50_ of the extracted BoNT/A was calculated about 4pg/mouse based on the result of the sequentially diluted BoNT/A intraperitoneal injection to mice. The protein activity of SNAP25 as a substrate was tested by incubation with BoNT/A. The result indicated that recombinant SNAP25 can be cleaved by BoNT/A.

### Identification of purity, titer, and specificity of anti-subA IgY

**Figure 2:**
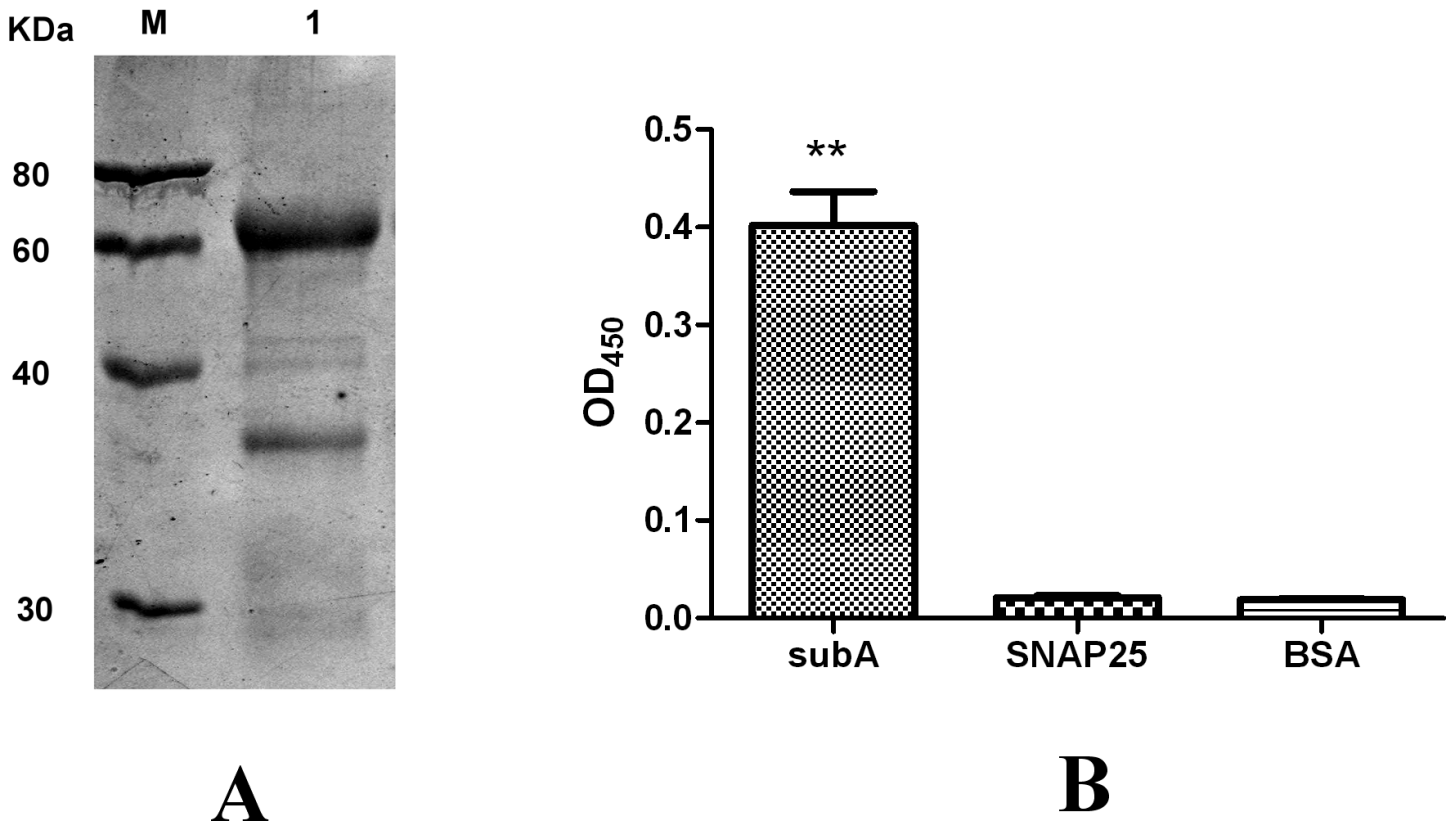
Purity and specificity analyses of purified anti-subA IgY. A) SDS-PAGE results of purified anti-subA IgY. M: protein molecular weight markers; Lane 1: purified IgY. B) ELISA result of Anti-subA IgY to subA, SNAP25, and BSA. *: notable statistical significance compared with BSA (*p* < 0.01), #: no statistical significance compared with BSA (*p* > 0.05).

Anti-subA IgY was obtained by injecting laying hens with the synthesized peptide from SNAP25. The anti-subA IgY was prepared from eggs by the water dilution method and purified with an IgY purification HiTrap HP column. The purified IgY was verified by SDS-PAGE, which showed 2 bands: the upper band at 65 kDa (heavy chain) and the lower band at 35 kDa (light chain). Their molecular masses matched the theoretically predicted ones (Fig. 2A).

After protein quantitation and titration, anti-subA IgY had a of 35.51 mg/ml and exhibited favorable binding activity at 1:320 000. Anti-subA IgY also cannot recognize the full-length SNAP-25, which has a spatial conformation; however, anti-subA IgY recognized the linear peptide cleaved only by BoNT/A only (Fig. 2B).

### Effects of buffer, time, and temperature on the endopeptidase assay

**Figure 3:**
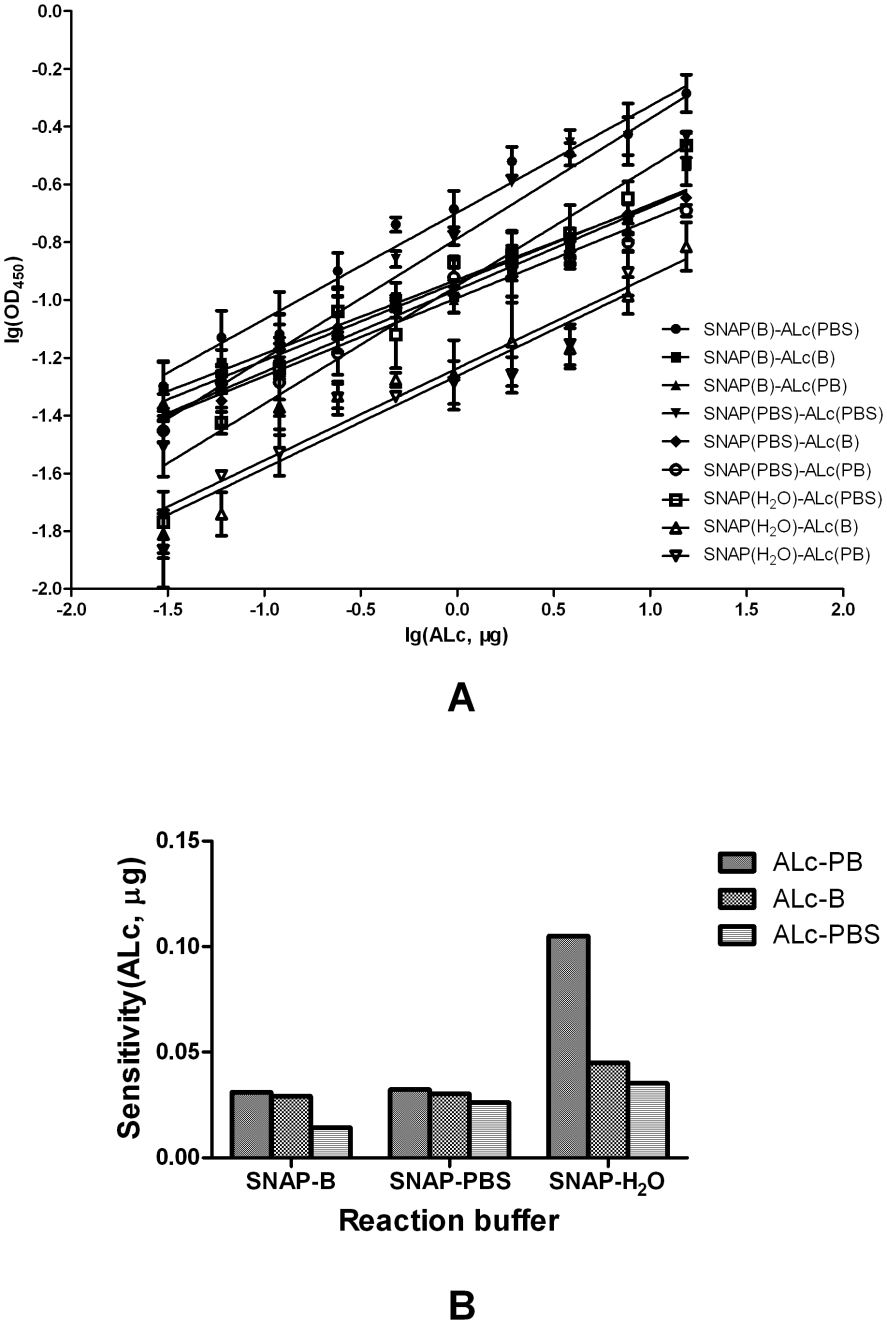
Effects of buffer in the ALc endopeptidase assay. A) The concentration–response relations between OD_450_ and ALc with different buffer combinations. B buffer: 0.02 M Na_3_PO_4_, 0.5 M NaCl, 0.4 M imidazole, pH 7.4; PBS buffer: 0.008 M NaH_2_PO_4_, 0.002 M Na_2_HPO_4_, 0.145 M NaCl, pH 7.2; PB buffer: 0.008 M d NaH_2_PO_4_, 0.002 M Na_2_HPO_4_, pH 7.2. B) Sensitivity of endopeptidase assay in different buffer combinations.

To determine the optimum conditions for the endopeptidase assay, the effects of buffer, time, and temperature were examined. First, the endopeptidase assay was performed with nine buffer combinations, namely, three SNAP25 buffers (PBS, B buffer, and H_2_O) with three ALc buffers (PBS, PB, and B buffers). To examine the dose–effect relationship of the endopeptidase assay of ALc in different buffers, gradient-diluted ALc was added to SNAP25 coated plates. The result of the endopeptidase assay was analyzed by ELISA and expressed in terms of the mean OD_450_ (Fig. 3A). The result showed that the OD_450_ reads and the quantity of ALc after log conversions presented linear concentration–response relations in different buffer combinations, with a correlation coefficient (*R* ^2^) > 0.9 (Table S1).

Sensitivity was calculated based on the average background values added to two times the standard deviation (2SD). The results showed that the sensitivity of the analysis system was the highest (*p* < 0.01, Fig. 3B) with SNAP25 in B buffer and ALc in PBS buffer. Thus, this buffer combination was used throughout the assay.

**Figure 4:**
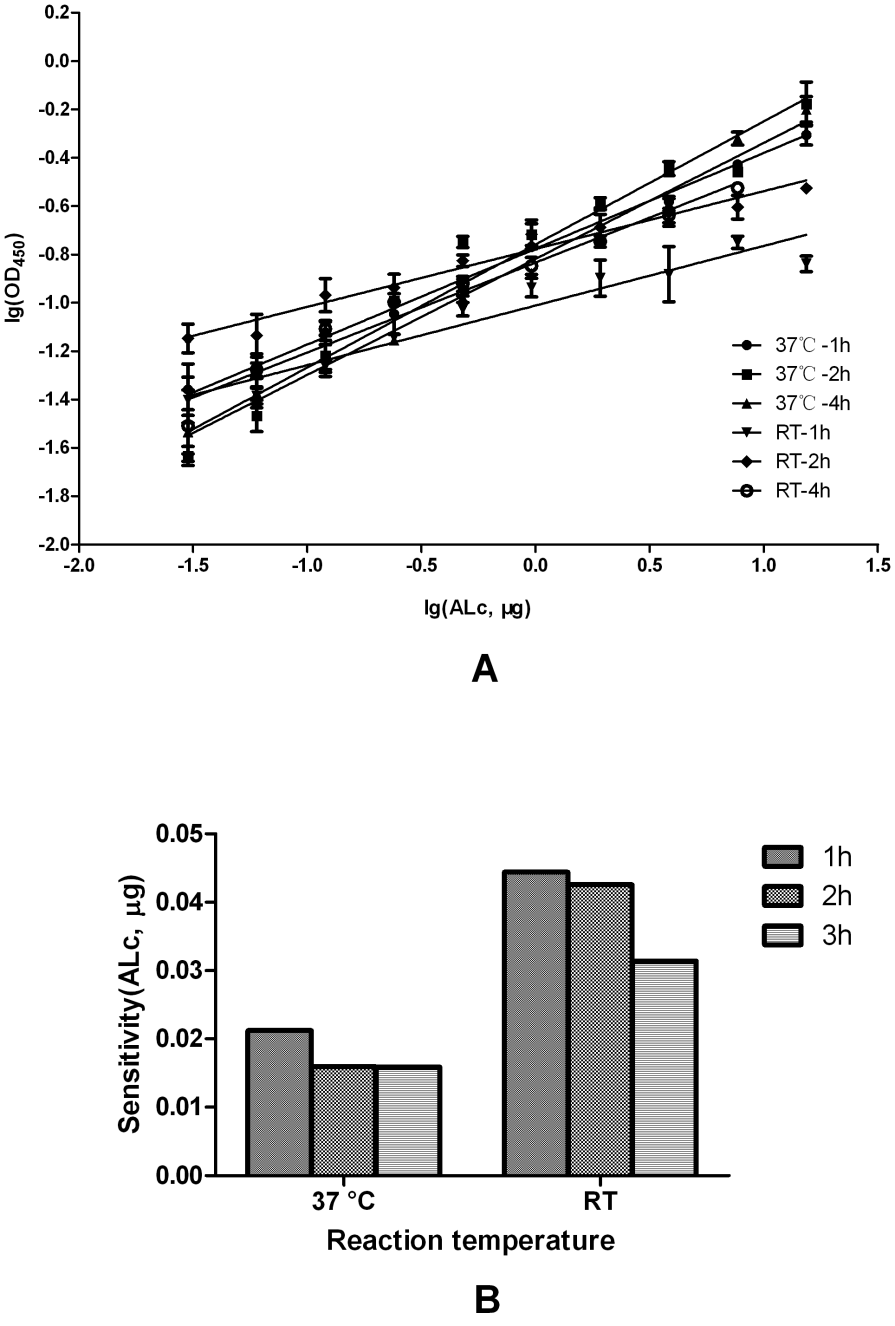
Effects of temperature and time in the ALc endopeptidase assay. A) Concentration–response relations between OD_450_ and ALc with different reaction temperatures and time combinations. B) Sensitivity of the endopeptidase assay at different temperature and time combinations.

The endopeptidase assay was also performed with different time and temperature combinations, which also showed linear concentration–response relations (Fig. 4A and Table S2). Based on the same analysis as the that on buffer effect, we found that the sensitivity of the endopeptidase assay at 37 °C was better than that at RT (25 °C; *p* < 0.01) (Fig. 4B). Whether this result reflected the thermal conformational stability of ALc and SNAP25 was unclear. However, the reaction time from 1 h to 4 h did not affect the assay (Fig. 4B).

Therefore, the assay with SNAP25 in B buffer and ALc in PBS buffer at 37 °C for 1 h was the optimum condition of the endopeptidase assay and was thus used in subsequent experiments.

3.4 LOD and LOQ for BoNT/A of the method

**Figure 5:**
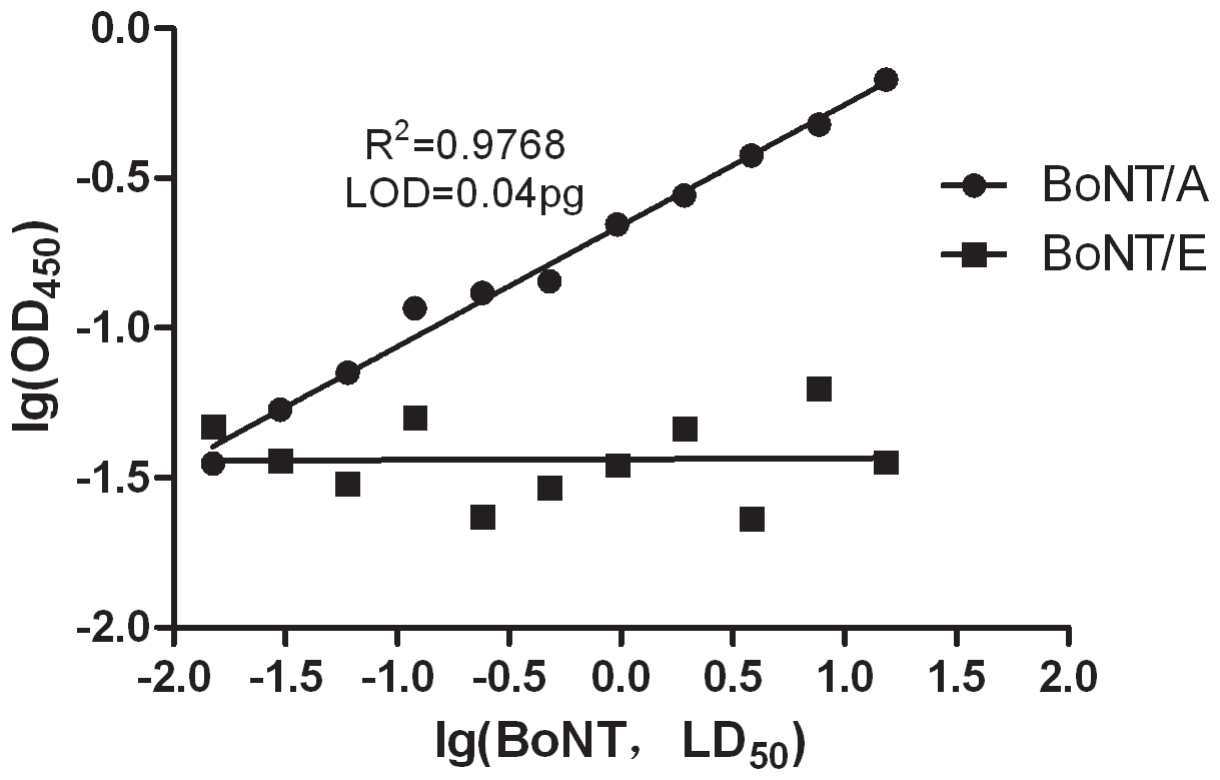
Detection and quantitation of BoNT/A and BoNT/E using IgY antibody.

The endopeptidase assay was used under optimized conditions to detect BoNT/A standard preparation. The results (Table 1 and Fig. 5) showed that the OD_450_ reads and the concentration of BoNT after log conversions presented linear concentration–response relations. The correlation coefficient reached 0.99, and the calculated equation concentration–response relations were used to compute the BoNT/A concentration in the test sample.

**Table 1:** Detection of BoNT/A with different concentration by anti-subA IgY.

Concentration of BoNT/A (LD_50_)	OD_450_			Mean OD_450_	SD	CV (%)
0	0.022	0.03	0.027	0.026	0.004	15.35
0.015	0.038	0.029	0.04	0.036	0.006	16.43
0.03	0.043	0.056	0.063	0.054	0.01	18.79
0.06	0.06	0.083	0.072	0.072	0.012	16.05
0.12	0.108	0.127	0.115	0.117	0.01	8.24
0.24	0.123	0.144	0.127	0.131	0.011	8.49
0.48	0.153	0.119	0.162	0.145	0.023	15.68
0.96	0.218	0.21	0.237	0.222	0.014	6.26
1.92	0.272	0.295	0.266	0.278	0.015	5.51
7.68	0.401	0.504	0.542	0.482	0.073	15.13
15.36	0.627	0.7	0.697	0.675	0.041	6.12

The background mean added to twice the standard deviation gave the LOD of the endopeptidase assay [24], which was 0.01 mouse LD_50_ (0.04 pg BoNT/A). Based on CV < 16%, the LOQ was 0.12 mouse LD_50_ (0.48 pg BoNT/A).

The relationship between the detection results of BoNT/A standard preparation and purified ALc was analyzed (P < 0.01) . The result proved that ALc can be used as control instead of BoNT/A during the development and application of the method.

### Specificity of the method

The specificity of the method was determined by analyzing the result of gradient diluted BoNT/E incubated with coated SNAP25 and compared with BoNT/A. In contrast to BoNT/A, the BoNT/E concentration and the OD_450_ reads did not present a concentration–response relation (Fig. 5). No positive reading was obtained even though the BoNT/E concentration reached 15.36 LD_50_, indicating that the specificity of the assay was good.

### Detection of BoNT/A in milk or serum samples and calculation of the method precision

BoNT/A with three different concentrations (0.24 mouse LD_50_, 0.32 mouse LD_50_, and 0.48 mouse LD_50_) in reaction buffer, milk, or human serum samples were detected using the proposed method. The BoNT/A concentration in the different samples were calculated (Table 2). Then, the calculated recoveries as well as the intra- and inter-assay precisions were analyzed. The results indicated that the method had good accuracy and precision (88% < recovery < 111%; inter- and intra-assay CVs < 18%).

**Table 2:** Detection of BoNT/A in reaction buffer, milk or serum samples.

Sample	Concentration of BoNT/A (LD_50_)	Calculated concentration of BoNT/A (LD_50_)			Intra-M (LD_50_)	Intra-SD (LD_50_)	Intra-CV (%)	Recovery (%)	Inter-M (LD_50_)	Inter-SD (LD_50_)	Inter-CV (%)
B	0.24	0.2265	0.2707	0.2218	0.2397	0.027	11.3	99.9	0.2511	0.0344	13.70
M	0.24	0.2505	0.2039	0.2865	0.247	0.0414	16.8	102.9			
S	0.24	0.2455	0.2407	0.3141	0.2668	0.041	15.4	111.2			
B	0.32	0.3675	0.3372	0.3255	0.3434	0.0217	6.3	107.3	0.3156	0.0324	10.30
M	0.32	0.3198	0.2656	0.3198	0.3017	0.0313	10.4	94.3			
S	0.32	0.3372	0.2919	0.2759	0.3017	0.0318	10.5	94.3			
B	0.48	0.4536	0.4397	0.3994	0.4309	0.0281	6.5	89.8	0.4291	0.0499	11.60
M	0.48	0.4823	0.3431	0.4679	0.4311	0.0765	17.8	89.8			
S	0.48	0.3801	0.4897	0.4059	0.4252	0.0573	13.5	88.6			

Sample B, M, S: BoNT/A mixed with reaction buffer, milk or human blood. Intra-M, Intra-SD, Intra-CV: the mean, standard deviation and coefficient of variabilityof calculated concentration of BoNT/A with same sample. Inter-M, Inter-SD, Inter-CV: the mean, standard deviation and coefficient of variabilityof calculated concentration of BoNT/A with different sample.

## Discussion

The mouse bioassay has been the standard for testing BoNT-containing samples for the past 30 years. However, this assay is time consuming, requires the use of many animals, and has poor repeatability. In recent years, the IgY antibodies produced in hens have been proven useful in many applications, including immunotherapy and immunodiagnostics [29], [30]. In the present study, we investigated the ability of IgY to detect and quantitate BoNT/A. First, we synthesized peptide subA:NKTRIDEANQ located N-terminal 10 a.a. from cleavage site of BoNT/A at SNAP25. Then, we used it as an antigen to immunize the leghorn hens. Finally, IgY antibody was prepared by the water dilution method and purified with an IgY purification HiTrap column.

The entire process of IgY antibody production was simple and convenient. Moreover, given that the antibodies were harvested from eggs, bleeding the animal was unnecessary. Therefore, this method of IgY production in hens favored animal welfare. The availability of a large amount of IgY from egg yolks makes it feasible to use as an antibody in toxin detection. The titer and specificity of IgY were confirmed by ELISA. Our data showed that the IgY titer steadily increased with increased booster vaccination. The anti-subA IgY titer reached 320 000 after four vaccinations, and remained stable for 1 year.

Several endopeptidase assays utilizing antibodies to 5, 7, or 8 a.a. of the exposed SNAP25 cleavage site have been developed [19]–[24]. However, these assays lack ideal reproducibility or sensitivity (0.13 ng/ml and 0.1–0.8 mouse LD_50_/ml, respectively) or suffer from the unwanted effects of albumin [19]–[23]. Jones et al. [24] developed an endpeptidase immunoassay with high sensitivity (0.01 LD_50_/ml), but it required more than 18 h to finish. In the current study, many changes were made to previous techniques to remove the step that may introduce errors into the assay, as well as to optimize the reaction condition and improve the sensitivity.

Full-length SNAP25 was recombinantly expressed and used as a substrate in this endopeptidase assay, different from previous reports using only part of the sequence of SNAP25 [10], [16], [17], [22]. The formation of natural conformation was easier than the formation of a partial sequence, which helped improve the sensitivity of the endopeptidase assay. The recombinant SNAP25 contained BoNT/A, BoNT/E, and BoNT/C1 cleaved sites; thus, it can further be used to detect BoNT/E and BoNT/C1. Different from other methods of BoNT/A detection [17]–[24], recombinant ALc instead of BoNT/A was used as a positive control in the immunoassay. This method can avoid operator exposure to BoNT/A, and remarakbly eliminate the effect of BoNT/A purity.

BoNT/A and ALc have different activities under different conditions [17], [24]. The reaction conditions in the current study were optimized to obtain better sensitivity. The results showed that the activities of SNAP25 and ALc varied with each buffer. In the Ni-NTA elution (B buffer) and PBS buffers, SNAP25 exhibited significantly higher activity than in ddH_2_O. In ddH_2_O, the residues at the cleavage sites may be more easily exposed without BoNT/A or ALc cleavage, which led to a higher background value and influenced the sensitivity. The study also showed that ALc had different stabilities with different buffers. Precipitation of ALc occurred in B buffer after storage at –20 °C, whereas ALc was relatively stable in PBS buffer. Finally, the sensitivity with optimized buffer was 7.3-fold higher than with SNAP25 in ddH_2_O and ALc in PB buffer. The results of reaction temperature optimization experiment showed that the sensitivity was higher at 37 °C than at RT. This result revealed that ALc had higher activity at 37 °C, consistent with previous reports on BoNT/A [17], [24]. However, the sensitivity of this method can also reach 0.1 pg at RT, indicating good temperature compatibility. The results of reaction time effect experiments showed that the method can achieve increased sensitivity with increased reaction time. However, the difference in sensitivity from 4 h to 1 h was not significant at 37 °C; thus, 1 h was chosen as the optimum response time.

The ability of the proposed method to detect BoNT/A in food or blood was investigated and analyzed. The proposed method also showed good quantitative accuracy (88% < recovery < 111%, inter- and intra-assay CVs < 18%) when used to detect and quantitate trace amounts of BoNT/A (0.24 mouse LD_50_) in milk or human serum. This result indicated that the complex ingredients of human serum or milk had little interference to the method. The accuracy of quantitation may further improve when the samples are pretreated, such as by concentration and desalination. According to previous reports [2], the LD_50_ of BoNT/A to humans is 0.1–1 ng/kg. Thus, 6 ng of BoNT/A may be lethal to an adult weighing 60 kg. The LOD of the proposed method was 0.01 mouse LD_50_ (0.04 pg). Therefore, the sensibility of this method was sufficient for the practical diagnosis of a patient. The method also only needed less than 3 h and did not rely on special equipment when the substrate (SNAP25) was coated in the well in advance, making it easy to use in clinical diagnosis.

In summary, a simple and rapid method of BoNT/A detection and quantitation was developed using anti-subA IgY antibody. High sensitivity (LOD  =  0.01 mouse LD_50_ and LOQ  =  0.12 mouse LD_50_) was obtained by optimizing the reaction substrate and conditions. Favorable specificity and anti-interference activity were achieved through BoNT/E and complex sample detection experiment. Therefore, the proposed method can be a valuable method of BoNT/A detection in food or for clinical diagnosis.

## Supporting Information

Table S1 the concentration-response relations between OD_450_ and ALc with different buffer combination.
(DOC)

Table S2 the concentration-response relations between OD_450_ and ALc with different reaction time and temperature combination.
(DOC)
