# Supplementary material for: Hypersensitive Detection and Quantitation of BoNT/A by IgY Antibody against Substrate Linear-Peptide
Source: PLoS One. 2013 Mar 21;8(3):e58908. doi: 10.1371/journal.pone.0058908 (PMC3605418; doi:10.1371/journal.pone.0058908)
Supplement: Table S1 — the concentration-response relations between OD450 and ALc with different buffer combination. (DOC) [file pone.0058908.s001.doc]

**Table S1 the concentration-response relations between OD_450_ and ALc with different buffer combination**

|  | | ALc buffer | | |
| --- | --- | --- | --- | --- |
|  |  | B buffer | PB buffer | PBS buffer |
| SNAP25 buffer | B buffer | y = 0.2574x - 0.9294 | y = 0.2701x - 0.939 | y = 0.3706x - 0.6965 |
|  |  | R^2^ = 0.966 | R^2^ = 0.9709 | R^2^ = 0.9824 |
|  | H_2_O | y = 0.3192x - 1.2363 | y = 0.3207x - 1.2625 | y = 0.4097x - 0.9501 |
|  |  | R^2^ = 0.9076 | R^2^ = 0.9152 | R^2^ = 0.9326 |
|  | PBS buffer | y = 0.2844x - 0.9645 | y = 0.2768x - 0.9891 | y = 0.3979x - 0.7981 |
|  |  | R^2^ = 0.9823 | R^2^ = 0.9747 | R^2^ = 0.9642 |

x: lg(ALc, μg), 0.03 μg < [Alc] < 15.36 μg; y: lg(OD_450_)
