# Supplementary material for: Hypersensitive Detection and Quantitation of BoNT/A by IgY Antibody against Substrate Linear-Peptide
Source: PLoS One. 2013 Mar 21;8(3):e58908. doi: 10.1371/journal.pone.0058908 (PMC3605418; doi:10.1371/journal.pone.0058908)
Supplement: Table S2 — the concentration-response relations between OD450 and ALc with different reaction time and temperature combination. (DOC) [file pone.0058908.s002.doc]

**Table S2 the concentration-response relations between OD_450_ and ALc with different reaction time and temperature combination**

|  | | Reaction time | | |
| --- | --- | --- | --- | --- |
|  |  | 1 h | 2 h | 4 h |
| Reaction  temperature | 37 °C | y = 0.3873x - 0.7814 | y = 0.4847x - 0.7734 | y = 0.4923x - 0.8101 |
|  |  | R^2^ = 0.9918 | R^2^ = 0.9593 | R^2^ = 0.9892 |
|  | 25 °C | y = 0.2472x - 1.0101 | y = 0.2393x - 0.8488 | y = 0.3719x - 1.057 |
|  |  | R^2^ = 0.9454 | R^2^ = 0.9696 | R^2^ = 0.9701 |

x: lg(ALc, μg), 0.03 μg < [Alc] < 15.36 μg; y: lg(OD_450_)
